# Efficacy and safety of ultrasound-guided implantation of fiducial markers in the liver for stereotactic body radiation therapy

**DOI:** 10.1371/journal.pone.0179676

**Published:** 2017-06-21

**Authors:** So Hyun Park, Hyung Jin Won, So Yeon Kim, Yong Moon Shin, Pyo Nyun Kim, Sang Min Yoon, Jin-hong Park, Jong Hoon Kim

**Affiliations:** 1 Division of Abdominal Radiology, Department of Radiology and Research Institute of Radiology, Asan Medical Center, University of Ulsan College of Medicine, Songpa-Gu, Seoul, KOREA; 2 Department of Radiology, Gachon University, Gil Medical Center, Guwol-dong, Namdong-gu, Incheon, Korea; 3 Department of Radiation Oncology, Asan Medical Center, University of Ulsan College of Medicine, Songpa-Gu, Seoul, KOREA; Yonsei University College of Medicine, REPUBLIC OF KOREA

## Abstract

**Objective:**

Stereotactic body radiation therapy (SBRT) for the treatment of a malignancy in the liver requires the perilesional implantation of fiducial markers for lesion detection. The purpose of this study is to evaluate the efficacy and safety of ultrasound (US) -guided marker implantation for SBRT.

**Methods:**

We retrospectively reviewed 299, US–guided, intrahepatic fiducial markers implanted in 101 patients between November 2013 and September 2014. SBRT-planning CT images were analyzed to determine the technical success of the implantation, the mean distance between the tumor margin and the marker, with the ideal location of fiducials defined as the distance between a marker and a tumor less than 3 cm and the distance between markers greater than 2 cm according to the tumor conspicuity seen on gray-scale US and the artifact obscuring tumor margins. We also evaluated procedure-related major and minor complications.

**Results:**

Technical success was achieved in 291 (97.3%) fiducial marker implantations. The mean distance between the tumor and the marker was 3.1 cm (S.D., 2.1 cm; range, 0–9.5 cm). Of 101 patients, 72 lesions (71.3%, 2.2 ± 1.0 cm; range, 0–3.0 cm) had fiducial markers located in an ideal location. The ideal location of fiducials was more common in visible lesions than in poorly conspicuous lesions (90.2% vs. 52.0%, *P* < 0.001). Seventeen markers (5.8%) developed beam-hardening artifacts obscuring the tumor margins. There were no major complications, although 12 patients (11.9%) developed minor complications.

**Conclusions:**

US-guided implantation of fiducial markers in the liver is an effective and safe procedure with only rare complications.

## Introduction

SBRT is a highly sophisticated, image-guided, radiation therapy. It uses multiple, photon beams that intersect at a stereotactically determined target, and therefore emits higher doses of radiation delivery to the tumor while sparing surrounding normal tissue [1]. This radiation therapy technique is useful as an ablative treatment for small tumors in various organs when other treatment options are not available [2,3]. This treatment is also effective for local control of both hepatocellular carcinoma (HCC) and liver metastases [4–7]. As the liver is very radiosensitive, accurate targeting of the tumor while salvaging normal hepatic parenchyma is crucial in order to prevent radiation-induced liver injury [8–10].

There are some technical difficulties when SBRT is applied to the liver. The liver is one of the organs moving continuously caused by respiration. It lacks inherent contrast between normal parenchyma and tumor, especially on nonenhanced CT which is commonly used during SBRT. The perilesional implantations of fiducial markers are helpful for enhancing the treatment accuracy of SBRT. Fiducial markers for SBRT are generally introduced under US guidance.

However, hepatic lesions are not always clearly visible on US due to the lack of echogenicity difference or a suboptimal sonic window resulting from heterogeneous liver parenchyma or their deep position [11–13]. While previous studies [14,15] have reported the efficacy and safety of US-guided, fiducial marker implants in the liver, the number of these studies is limited due to the small number of patients. Moreover, they did not specifically mention the efficacy of US-guided, fiducial marker insertion in poorly conspicuous lesions. Therefore, the purpose of this study is to assess the efficacy and safety of US-guided, marker implantation in the liver for SBRT, considering the conspicuity of lesions on US.

## Materials and methods

This retrospective study was approved by Asan Medical Center Institutional Review Board, and written informed consent was waived.

### Study patients

We retrospectively identified 108 patients who had undergone percutaneous, US-guided, fiducial marker implantations for SBRT of the liver between November 2013 and September 2014. The patients were evaluated by the radiation oncologists in order to determine their suitability for SBRT in the liver according to the following criteria [4]: (1) the tumor cannot be treated by surgery due to liver cirrhosis, insufficient remnant liver volume or patient refusal of surgery; (2) the tumor was located in the liver surface near large vessels or a bile duct or at the top of the liver where percutaneous, local ablative therapies cannot be performed; (3) the tumor was confined to the liver without extrahepatic metastases; (4) liver function was classified as Child-Pugh class A or B; (5) an adequate functional remnant liver was evident (> 700 cc); (6) there was a sufficient distance (> 2 cm) between the tumor and adjacent organs at the duodenum, stomach, colon or spinal cord; and (7) an incomplete response or unsuitable for transarterial chemoembolization (TACE). Among them, we excluded seven patients with multiple hepatic tumors, as it is difficult to determine the relationship between fiducial markers and index tumors. Therefore, 101 patients (mean age, 61.3 years; age range; 42–80 years) underwent implants of 299 fiducial markers. Liver lesions included 89 HCCs, metastases from colon cancer (n = 5), angiosarcoma (n = 1), ampulla of vater cancer (n = 1), endometrial cancer (n = 1), gall bladder (GB) cancer (n = 2), stomach cancer (n = 1), and primary serous papillary carcinoma of the peritoneum (n = 1) (Table 1).

**Table 1:** Patient demographics.

Characteristics		Value (n = 101)
Age (years)		
	Mean ± SD	61.3±9.2
	Range	42–80
Male/female		73/28
Liver cirrhosis, yes/no		81/20
Chronic liver disease, yes/no		86/15
Child-Pugh class*, A/B		82/19
Hepatitis etiology		
	Hepatitis B virus	70 (69.3)
	Hepatitis C virus	4 (4.0)
	Alcoholic liver disease	14 (14.0)
	None	15 (14.7)
Liver		
	HCC	89 (88.1)
	Colon cancer	5 (4.9)
	Angiosarcoma	1 (1.0)
	AOV cancer	1 (1.0)
	Endometrial cancer	1 (1.0)
	GB cancer	2 (2.0)
	Stomach cancer	1 (1.0)
	PSPCP	1 (1.0)
The longest tumor diameter, cm		
	Mean ± SD	2.0 ± 1.2
	Range	0.7–6.8
Location of tumor		
	segment 1	4
	segment 2	11
	segment 3	7
	segment 4	16
	segment 5	11
	segment 6	10
	segment 7	14
	segment 8	28
Portal vein invasion, yes/no		5/96

Data in parentheses are percentages. HCC (hepatocellular Carcinoma), AOV (ampulla of vater), GB (gall bladder), PSPCP (primary serous papillary carcinoma of the peritoneum)

* Child-Pugh class A includes patients without cirrhosis. Liver-segments were separated by the Couinaud classification.

### Fiducial marker implantation

All fiducial marker insertions were performed by four, abdominal radiologists, each in fellowship training with >50 cases of US-guided procedures. In our hospital, patients with an uncorrectable bleeding tendency (prothrombin time ratio < 50%, international normalized ratio (INR) >1.7, and platelet count < 50,000 cells/mm^3^) did not undergo this procedure. After reviewing the previous imaging studies of multiphase CT or contrast-enhanced, magnetic resonance image (MRI), the best percutaneous needle approach was planned and fiducial marker insertion was performed with real-time US guidance using a 1-5MHz convex probe (LOGIQ E9 (GE Healthcare, Waukesha, WI, USA). A gold seed (CIVCO Medical Solutions, Kalona, IA, USA) was used as a fiducial marker. Each fiducial marker was 5 mm long and 1 mm in diameter. Each was assembled on 20-cm syringes with an 18-gauge caliber using a 17-gauge guide. The tumor conspicuity was determined as whether the margin was visible or poorly conspicuous on gray-scale US. When a tumor was visible on gray-scale US, patients had three, fiducial markers implanted around the tumor using real-time US. If a tumor was inconspicuous on gray-scale US, the perilesional implants were performed with correlation to the vessel anatomy and tumor assessed by reviewing the previous CT or MRI and US or using real-time fusion imaging.

### Assessment of marker implantation

All CT images were interpreted in consensus by two, abdominal radiologists with five and 16 years, respectively, of clinical experience performing abdominal CT ([BLINDED], and [BLINDED]).

Technical success was defined as when all of the implanted fiducials were detected in the liver around the tumor on the planning CT of SBRT which was performed within the next month (mean ± standard deviation (SD), 7.8 ± 3.1 days; range, 0–21 days) following insertion of the fiducial markers and possible to perform SBRT. If a fiducial marker was not visible or was in an organ other than the liver on CT, it indicated that there had been migration of the fiducial marker and which was classified as technical failure. If an additional marker was required in order to perform SBRT because the marker had been inserted in an inappropriate site, it was also considered as technical failure.

**Fig 1:**
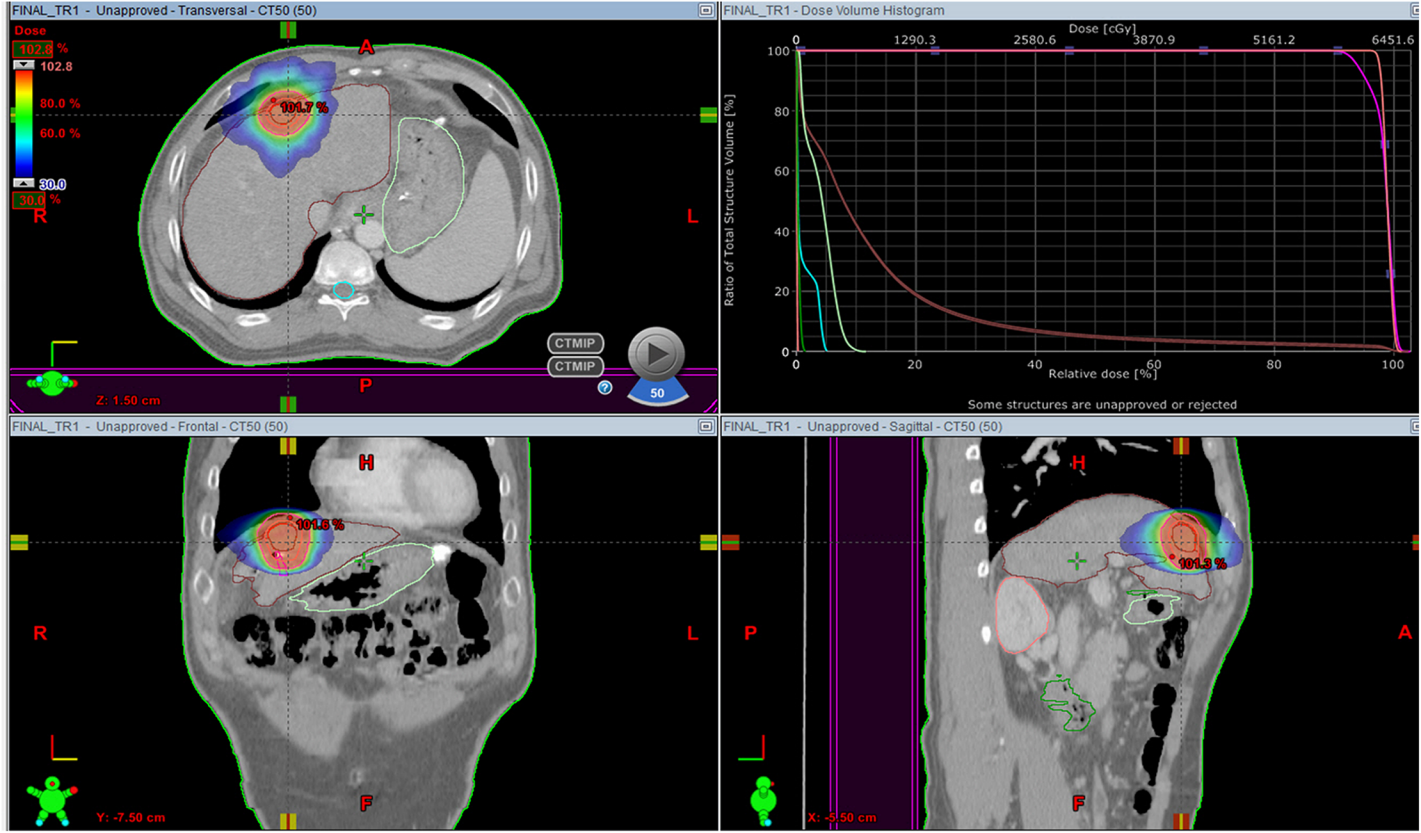
An example of radiotherapy planning using a respiratory-gated, volumetric-modulated, arc therapy technique for hepatocellular carcinoma in segment 3 of the liver.

**Fig 2:**
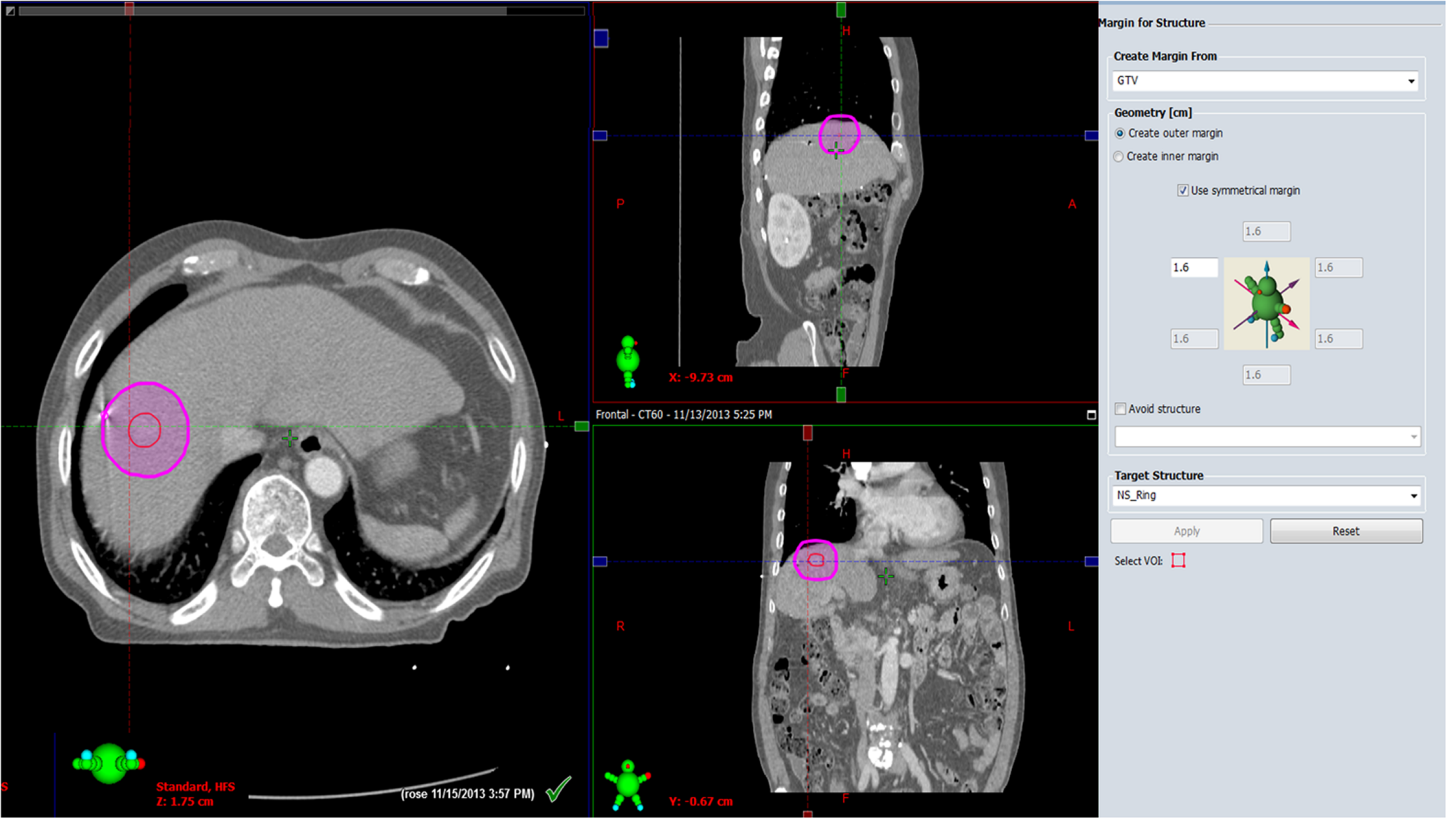
Calculating the distance semi-automatically between the tumor margin and a marker in 3-dimensional space creating a curved line parallel to the outer margin of the tumor using planning CT workstation. Firstly, draw a line along tumor margin. Second, create a curved line parallel to the outer margin of the tumor at marker site using workstation tool. And the distance between the tumor margin and a marker is automatically calculated as 1.6cm.

Tumor size (the longest tumor diameter) and location and the distance between the tumor margin and markers were evaluated on planning CT (Fig 1) using a workstation (Eclipse; Varian, Palo Alto, CA, USA). We calculated the shortest distance between the tumor margin and a marker in 3-dimensional space, creating a line parallel to the outer margin of the tumor (Fig 2) and compared that between visible and poorly conspicuous lesions on US. Modified from previous work [2,16] and after discussion with radiation oncologists in multidisciplinary conferences, the ideal location of fiducial markers was considered as the distance between a marker and a tumor of less than 3 cm and the distance between markers greater than 2 cm [2,16]. We evaluated the ideal location of fiducials according to the tumor conspicuity seen on US. The presence of beam-hardening artifacts caused by the markers was also assessed on CT.

Implanted, marker-related complications were evaluated on immediate post-procedural Doppler US in the patient’s electronic medical records (EMR) and on planning CT. Major complication was defined as those requiring interventional treatment or prolonged hospital admission (> 48 hours) [17]. Minor complication was defined as symptoms or signs which improved with conservative management.

### Statistical analysis

We evaluated the technical success and failure rates of the procedures. The relationship between markers and tumors was also assessed. Visible lesions and poorly conspicuous lesions on US were compared in terms of their clinical characteristics and assessment of marker implantation using the chi-square and student’s t test. The student’s t-test was used to evaluate the relationship between procedure-related bleeding and INR. Statistical analysis was performed using IBM SPSS Statistics for Windows Version 21.0 (IBM Corp, Armonk, NY, USA). A two-sided *P* value < 0.05 was considered statistically significant.

## Results

Of 299 fiducial implantations in the liver, technical success was achieved in 291 (97.3%) implantations (Table 2). The mean number of fiducial markers implanted per procedure was 2.88. Of the 101 patients, 97 had undergone three, marker implantations. Of those 97 patients, eight developed migration of one of the three markers. Four patients each underwent two, marker implantations for the following reasons: two patients had a 70,000/mm^3^ and a 75,000/mm^3^ platelet count, respectively. One patient was uncooperative during the procedure, and the other patient had a small amount of perihepatic ascites before the procedure. No marker migration occurred in those four patients with implantation of two markers each. In summary, eight markers (2.7%) developed migration. Of those eight, migrated markers, seven were not seen on the planning CT. One marker was found in the subcapsular area in which the lesion was located. However, no patient required additional fiducial marker insertion due to previous migration, as the radiation oncologists decided that radiation therapy could be performed without an additional marker.

**Table 2:** Technical success and complications in 101 patients.

Characteristics	Value (Total markers = 299)
Technical success	291/299 (97.3)
Marker migration	8/299 (2.7)
Complications	
Major	0
Minor	12/101 (11.9)

Data in parentheses are percentages.

**Fig 3:**
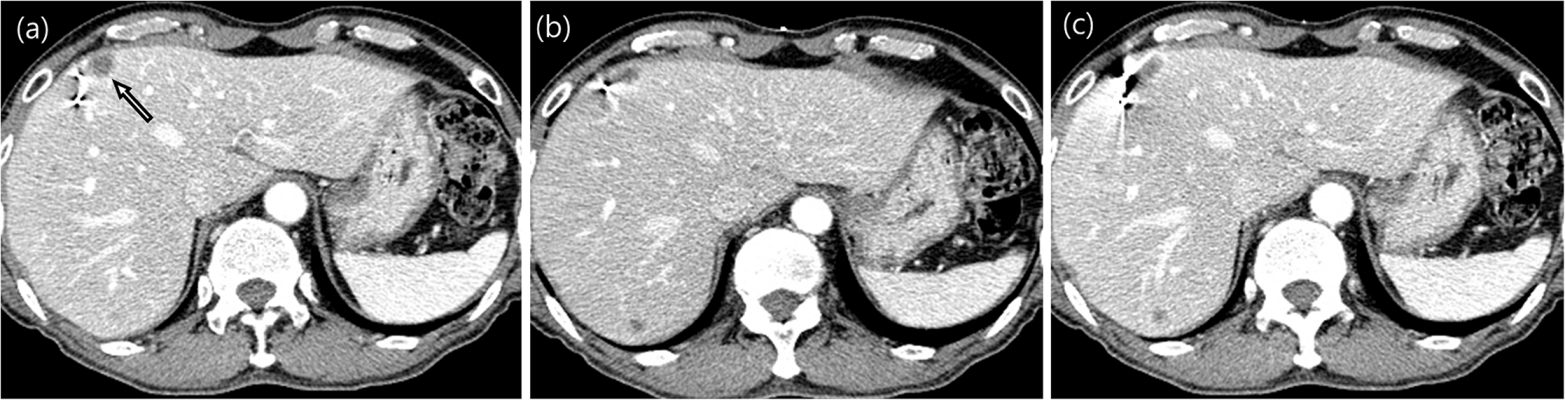
Beam hardening artifact created by the marker obscuring tumor margins. (**a**) Hepatic metastasis (arrow, the longest tumor diameter: 1.4 cm) from rectal cancer in segment 4 of the liver. (**b, c**) Beam-hardening artifact of the marker obscured the tumor margin. The distance between the tumor and the marker was 0.4 cm.

The mean overall tumor diameter was 2.0 cm (± 1.2 cm; range, 0.7–6.8 cm). The mean distance between the tumor margin and the markers was 3.1 cm (± 2.1 cm; 0–9.5 cm). Fifty-one lesions were well-visualized on US, while 50 lesions were in poor conspicuity on US. In 48 of these 50 lesions, the marker placement was performed under US guidance according to their correlation with the vessel anatomy and tumor using previous CT or MRI scans and US. Two poorly conspicuous lesions seen on gray-scale US became more clearly identifiable on fusion imaging. There was a significant difference in the mean distance between the tumor and the marker when comparing well-visualized lesions with poorly conspicuous lesions on US (*P* = 0.012, Table 3). The mean distance between the tumor and the marker in poorly conspicuous lesions on US (4.0 ± 3.3 cm; range, 0.8–9.5 cm) was significantly longer than that in visible lesions (2.6 ± 1.3 cm; range, 0–4.3 cm). In the 101 patients, the ideal location of fiducial markers was in 72 lesions (71.3%, 2.2 ± 1.0 cm; range, 0.7–3.0 cm). The ideal location of fiducials was more common in visible lesions than in poorly conspicuous lesions on US (46 [90.2%] vs. 26 [52.0%], *P* < 0.001). Seventeen markers (5.8%) developed a beam-hardening artifact and thus making the evaluation of the mass more difficult in 16 patients (Fig 3). In these patients, the mean distance between the tumor and the marker was 0.4 cm (± 0.4 cm, 0–1.0 cm) and the mean overall tumor diameter was 1.4 cm (± 0.7 cm; range, 0.7–2.7 cm). Three fiducial markers were inserted into each of these tumors.

**Table 3:** Comparison of visible and poorly conspicuous lesions on gray-scale US.

Characteristics		Visible (n = 51)	Poorly conspicuous (n = 50)	*P* value
Age (years)				0.823
	• Mean ± SD	61.5±8.7	61.1±9.8	
	• Range	44–80	42–79	
Male/female		34/17	39/11	0.267
Liver cirrhosis, yes/no		36/15	45/5	0.023
The longest tumor diameter, cm				0.203
	• Mean ± SD	2.2 ± 1.2	1.9 ± 1.3	
	• Range	0.8–5.5	0.7–6.8	
Location of tumor				0.061
	• segment 1	2	2	
	• segment 2	9	2	
	• segment 3	2	5	
	• segment 4	12	4	
	• segment 5	6	5	
	• segment 6	5	5	
	• segment 7	5	9	
	• segment 8	10	18	
Portal vein invasion, yes/no		3/48	2/48	1
Ideal location of fiducials, yes/no		46/5	26/24	<0.001
Mean distance between the tumor and the marker		2.6 ± 1.3	4.0 ± 3.3	0.012
Migration, yes/no		4/47	4/46	1
Complications, yes/no		9/42	3/47	0.122
Metal artifact, yes/no		9/42	6/44	0.577

**Fig 4:**
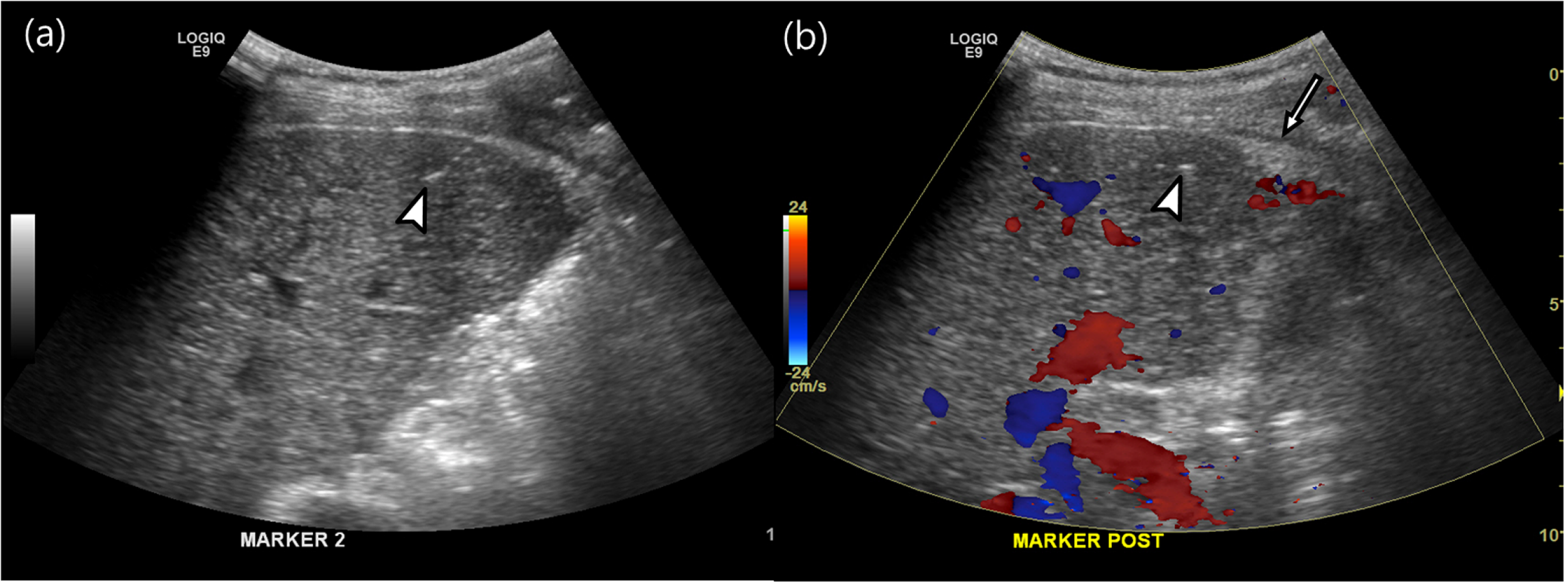
Minor complications. A 43-year-old male with hepatocellular carcinoma who underwent fiducial marker implants (arrowheads, **a-b**). After the procedure, a small, echogenic lesion (**b**) developed in the subcapsular portion of the liver, thus suggesting subcapsular hematoma (*arrow*).

There were no major complications in any patients, although 12 patients developed minor complications. One patient suffered from pain after the procedure and which improved during managed overnight observation. One patient had a subcapsular hematoma but did not require any intervention (Fig 4). Ten patients developed immediate post-procedural bleeding along the needle tract, as seen on color Doppler US. Following manual compression, all of these patients’ abnormal blood flow disappeared, as seen on color Doppler US. Eleven patients with minor bleeding complications had 1.1±0.1 INR (mean ± SD, range, 0.9–1.4). There was no significant difference in the INR between those patients with bleeding complications and those without bleeding complications (*P* = 0.131).

## Discussion

Our results indicated that US-guided implantation of fiducial markers in the liver had a high technical success rate and few complications. Our technical success rate was 97.3% (291/299), and the migration rate was 2.7% (8/299). Consistent with our results, previous studies have reported that the technical success of US-guided marker implantations in the liver was as high as 97% and the migration rate was 0–2.2% [2,14,18]. One study reported the cause of migration to be a tumor located in the adjacent hepatic capsule [14]. In our study, one marker was placed in the perihepatic space in which the lesion was located in the subcapsular area of segment 3. The seven, remaining migrations were not associated with tumor located in the adjacent hepatic capsule. Seven markers were not detected on planning CT which indicated migration. We might, therefore, suggest that the marker had been implanted in an inappropriate site such as a vessel or peritoneal cavity and had developed migration.

We had no major complications and the minor complication rate was 11.9% (12/101). These results agree with those of previous studies which have reported a 0–3% major complications rate and a 3–17% minor complications rate [2,14,18]. In our study, among 12 patients, one patient developed subcapsular hematoma in the perihepatic space and ten (9.9%, 10/101) patients had immediate, post-procedural bleeding along the needle tract, as seen on Doppler US. With regard to the risk factors of minor complications, there was no relationship between minor bleeding and the INR. We suspect that these results might be due to the fact that patients with severely abnormal INR values were excluded from our study.

US guidance had limitations because the lesions were poorly conspicuous either due to heterogeneous liver parenchyma or deeply located lesions in 50 of the 101 lesions in our study. The mean distance between the tumor margin and the marker in the poorly conspicuous lesions seen on US was significant greater than that in the visible lesions. The ideal location of the fiducial markers was also significantly higher in the visible lesions than in the poorly conspicuous lesions seen on US. We used fusion imaging of real-time US with CT when a tumor was inconspicuous on gray-scale US and the radiologist considered that it would be helpful to detect the lesion using fusion imaging. Two, poorly conspicuous lesions seen on gray-scale US became more clearly identifiable on fusion imaging of real-time US with CT. However, this study had very small numbers of patients using fusion imaging and limited evaluation. Contrast-enhanced US is also useful for interventional procedures in order to improve the contrast between liver parenchyma and tumor [19–21]. Fusion imaging of real time US with CT and contrast-enhanced US may also be effective in poorly conspicuous lesion on gray-scale US and, further studies are needed to evaluate its feasibility. However, no patient had additional fiducial marker insertion because the treatment was not feasible.

Another point raised by our study is at least a 1-cm distance between the fiducial marker and the tumor was recommended in order to avoid tumor-margin blurring. Fiducial markers can develop artifacts and obscure margins of the tumor, especially in small lesions. This is important as an indistinct tumor margin offers only limited evaluation of a tumor on planning and follow-up CT studies. In our study, 17 markers (5.8%) developed a beam-hardening artifact obscuring tumor margins in 16 patients. In our patients with artifacts obscuring the tumor margin, the mean overall tumor diameter was 1.4 cm and the mean distance between the tumor and the marker was 0.4 cm (± 0.4 cm, 0–1.0 cm). Kothary et al. reported that if the tumor diameter is less than 2 cm, a marker inserted into the tumor may obscure the tumor margin [18].

Optimal positioning of fiducials in relation to a lesion might vary according to the equipment used for SBRT. When performing fiducial implantations for SBRT using CyberKnife, it is advised to maintain a minimum spacing of 15 mm and a minimum 15° angle between the fiducials [18,22]. Fiducial implantation with CyberKnife uses real-time tracking of tumors during the entire treatment cycle during which radiographic landmarks are obtained using two, fixed x-ray sources arranged orthogonally to the patient [16,18]. Although radiologic landmarks in LINAC SBRT are obtained by cone-beam CT, there is no specific instruction regarding the minimum spacing or angle between fiducials. On the other hand, fiducial implantations may develop the metallic artifact of markers obscuring the tumor margin when there was less than a 1-cm distance between them. Therefore, an appropriate distance between the marker and the lesion is required in order to evaluate the tumor margin and perform SBRT.

Our study had several strengths compared with previous, related studies. We focused on US-guided fiducial marker insertion in liver lesions and with a relatively large case number. Moreover, as we calculated the distance between the tumor margin and a marker in 3-dimensional space, we could more accurately measure the distance. We found that an appropriate distance between the fiducial marker and the tumor is useful in order to avoid unnecessary artifacts. In our study, we intended to evaluate feasible marker insertion in the liver from a more practical aspect regarding cases of poorly conspicuous lesions seen on US.

Our study does have limitations. First, our study results were limited by the retrospective design of our study. Although we recruited the patients based on our inclusion and exclusion criteria, a selection bias may have resulted from the study design. As we reviewed patients’ symptom based on EMR, there is a possibility that a few symptoms were omitted from the records. Second, there were a relatively large number of patients with HCC compared with the number of patients with metastasis. We did not evaluate the differences between HCC and metastasis regarding procedure-associated complication and migration rate. Finally, we did not assess the treatment response of tumors regarding the distance between a marker and a tumor. There was no patient who had additional fiducial marker insertion because the treatment was not feasible whether or not there was ideal location of the fiducial markers. However, it may be clinically useful to evaluate the ideal location of fiducial markers correlated with the treatment response of the tumor.

In conclusion, our study results showed that US-guided implantation of fiducial markers in the liver for stereotactic body radiation therapy had a high technical success rate and with rare complications. However, it is technically difficult to implant fiducial markers in the ideal location when a tumor is poorly conspicuous on gray-scale US. At least a 1-cm distance between the fiducial marker and the tumor is recommended in order to avoid tumor margin blurring caused by the beam-hardening artifact.

## Abbreviations

HCC: hepatocellular carcinoma
AOV: ampulla of vater
GB: gall bladder
PSPCP: primary serous papillary carcinoma of the peritoneum
SBRT: stereotactic body radiation therapy
